# Formulation and evaluation of the efficacy of an artificial larval diet for rearing various species of flies under laboratory conditions

**DOI:** 10.3389/finsc.2025.1630472

**Published:** 2025-08-08

**Authors:** Francesco Defilippo, M. Denise Gemmellaro, Annalisa Grisendi, Vito Tranquillo, Antonio Lavazza, Michele Dottori, Ana Moreno

**Affiliations:** ^1^ Department of Virology, Istituto Zooprofilattico Sperimentale della Lombardia e dell’Emilia-Romagna, Brescia, Italy; ^2^ Department of Biological Sciences, Kean University, Union, NJ, United States

**Keywords:** Calliphoridae, artificial diet, insect rearing, black soldier fly, Muscidae

## Abstract

The larvae of five dipteran species were reared on artificial diets under controlled laboratory conditions. Usually, these species complete their life cycles in perishable, filthy, unhygienic, and foul-smelling natural diets, which hinder rearing work and affect the laboratory environment. More importantly, these unaltered foods do not allow for true conformity in rearing protocols. The addition of a standard artificial diet to rearing protocols would make it possible to conduct ecological, biological, and forensic investigations with greater accuracy and precision. To address this problem, we formulated a new artificial diet for larvae and tested its performance on five different fly species (*Calliphora vicina, Lucilia sericata, Sarcophaga argyrostoma, Musca domestica*, and *Hermetia illucens*). We compared the development of larvae reared on an artificial diet with that of larvae reared on beef liver and pig muscle. The results showed no differences in development time between the two groups. However, our results showed that the artificial diet facilitated the rearing of flies for forensic and medical purposes by standardizing the nutritional value of the diet, improving laboratory conditions, and providing a more hygienic and cost-effective food substrate.

## 1 Introduction

Rearing insects is an essential part of entomological research. The use of artificial diets has allowed researchers to conduct studies in the fields of biological control (1); sterile insect technologies, feed for other animals (2); bioreactors for the production of pharmaceuticals (3) and other recombinant proteins; food for people (4, 5), and in recent years, in forensic entomology (6–8). This last branch of entomology involves studying the life cycles of insects that are found on decaying human remains and can be used to determine the date and cause of death (9).

Families of insects belonging to the order Diptera, which are of forensic importance, are numerous and differ from each other in their ecological characteristics. In forensic entomology, it is important to estimate the postmortem interval (PMI) (10). A reliable estimate of PMI requires a proven assessment of insect development rates under controlled laboratory conditions. Because breeding conditions can considerably influence the growth and development of larvae (11), such influences should be considered appropriately to avoid miscalculation of the PMI.

Most studies of larval growth rates have focused on factors such as temperature (12–14), larval aggregation and crowding, and drug effects (15, 16). There has been considerable variation in food substrates in these studies, including mammalian muscle, ground beef, mouse/rat carcasses (17, 18), fish, or even artificial diets containing components such as whole milk powder, baking powder, and wheat germ. However, these food substrates have proven to be efficient for the breeding of only some dipterans of forensic interest and, therefore, are not functional for the possible standardization of breeding in the laboratory (19).

Currently, forensic laboratories rely on natural materials, such as liver or other meat products, to rear these insects (8). These types of food sources are impractical for crime laboratories as they are usually kept frozen before use and are not shelf-stable, presenting an additional challenge in the collection of entomological evidence (6). The use of these materials results in noxious odors and potential health hazards associated with their unclean and nonstandard nature. Furthermore, because of their different nutritional contents, the use of these substrates causes high variability in insect development, which affects the subsequent interpretation of the data (20).

There is great interest in necrophagous arthropods, both in relation to decomposition ecology and forensic investigations (21). However, the lack of standardization of laboratory diets, feeding, and rearing protocols makes it difficult to obtain accurate and univocal data from behavioral, medical, and forensic entomology studies (22). As an alternative, employing an artificial diet that can be stored long-term in a refrigerator with a specific nutritional value, and with performance that is similar to that of the diets currently in use, would provide a simple solution to this problem.

In this study, we proposed a new artificial diet to replace the natural diets commonly used for rearing forensically important flies. We tested this diet on two blow fly species (Diptera: Calliphoridae; *Calliphora vicina, Lucilia sericata*), one muscid species (Diptera: Muscidae; *Musca domestica*), one flesh fly species (Diptera: Sarcophagidae; *Sarcophaga argyrostoma*), and the black soldier fly (Diptera: Stratiomyidae; *Hermetia illucens).* These flies usually feed on fermenting or rotting organic materials of either animal or vegetable origin, including human corpses. Therefore, they are important indicators in forensic investigations (23).

## 2 Materials and methods

Adult *Lucilia sericata, Calliphora vicina, Musca domestica, Sarcophaga argyrostoma, and Hermetia illucens* individuals were initially collected from Reggio Emilia in northern Italy (Lat. 44°42′32.34″N, Long. 10°37′36.66″E) using aerial traps baited with pork meat and beef liver. Adults were identified based on their morphological characteristics, as previously described (24). Approximately 100–150 individuals were placed in insect dorms of 30 cm × 30 cm × 30 cm with water, granulated sucrose (10:1 ratio), and 100 g of fresh beef liver in a Petri dish for oviposition.

### 2.1 Formulation of the artificial diet

The ingredients of the artificial diet were powdered milk; casein; soy flour; yeast; and agar, which was used as the gelling agent. The diet was prepared by weighing and mixing all of the dry ingredients as follows: 50 g of soy flour, 10 g of casein, 15 g of powdered milk, and 15 g of Saccharomyces cerevisiae yeast powder We dissolved 10g of agar in 200ml of distilled water.

All ingredients were mixed thoroughly and added to the agar solution. The mixture was then transferred to a Petri dish (90 mm diameter). Propionic acid was nebulized into the mixture as a mound inhibitor. The diets were stored in a refrigerator until further use. Preliminary tests were conducted to determine the appropriate box size, number of eggs per box, and diet for optimal larval development and survival. We have tested the number of specimens per box to avoid overcrowding phenomena, and the pH of the artificial diet. The pH of the final composition is between 6.8 and 7.5, this range is optimal for larval growth as it positively influences palatability and the activation of enzymes involved in digestion (4)

### 2.2 Experimental design

The different types of food used in the study were beef liver, pig muscle, and the artificial diet. All experiments were performed in triplicate to avoid the potential effects of other factors, such as temperature and humidity. The experiments were conducted under a photoperiod of 16 h/8 h light/dark, 25 ± 1°C, and 70% ± 5 relative humidity.

Each experiment involved three containers (liver, meat, and artificial diet) containing 100 g of each food type. To complete the larval stage, the food substrate was added *ad libitum*.

For the deposition of eggs of Calliphoridae and larvae of Sarcophagidae, liver was used as a substrate, while for Muscidae and Stratiomyidae, the Gainesville diet (25) was used.

In each container, 200 eggs were added, and the containers were placed in a 2 L plastic box.

All three experiments were performed simultaneously. Once pupated, 10 pupae were randomly collected and weighed to determine the mean individual pupal weight. Developmental, larval, and pupal times, pupal weight, and the percentage of adult emergence were compared between diets. The emergence of adults, pupal weight, and completion of the larval and pupal stages were recorded daily.

### 2.3 Statistical analysis

For each species, the effects of time and diet on weight (expressed in µg) were analyzed using a general linear mixed effects model, with the “replicate” variable as a random effect and the Gaussian weight distribution as a link function, using the lmer function of the lme4 package (26). There is a growing consensus within the biomedical scientific community that a significant/non-significant dichotomy based on a predetermined p-value cutoff should not be used for the interpretation of results. As such, we focused on the extent of the estimated treatment effect and its uncertainty, and the confidence intervals of the model estimates were interpreted as compatibility intervals, as described by Artheinm (27), accordingly, no null-hypothesis significance testing was performed. If an interval includes zero, this suggests that a null effect is plausible.

## 3 Results

Data collected for six life parameters—larval and pupal time, pupal weight, percentage of adult emergence, death rate, and total developmental period—were compared for the three diets and five species. The “adequacy” of an insect rearing substrate is determined by several factors, including its physical and chemical composition and its ability to support growth and development. For this reason, the suitability of the three diets was determined using an index incorporating growth, development, fecundity (indicated by pupal weight), and survival as empirical factors, as described by Seth and Sharma (28) (**Table 1**).

**Table 1:** Growth and development of *Hermetia illucens* (a), *Musca domestica* (b), *Calliphora vicina* (c), *Lucilia sericata* (d), and *Sarcophaga argyrostoma* (e) with three different diets.

Specie	Larval diet	Larval (days)	Pupal (days)	Pupal weight (g)	% Emergents	Death rate (%)	Develop. period (days)	^1^Growth index	^2^Adequacy index
*H. illucens*	Artificial diet	28 ± 0.7	6	0.14 ± 0.002	87	13	34 ± 3	2.6	0.5
	Beef liver	45 ± 0.3	18.3 ± 0.6	0.15 ± 0.03	32	68	63 ± 2	0.5	0.1
	Pig muscle	37 ± 0.2	7.5 ± 1	0.19 ± 0.05	64	36	45 ± 2	1.4	0.3
*M. domestica*	Artificial diet	5 ± 0.2	4	0.03 ± 0.002	98	2	9	10.9	0.6
	Beef liver	5 ± 0.3	3.6	0.02 ± 0.001	92	8	9	10.2	0.4
	Pig muscle	4.6	3	0.03 ± 0.001	98	2	9	10.9	0.6
*C. vicina*	Artificial diet	7 ± 0.2	6	0.05 ± 0.002	84	16	13	6.5	0.6
	Beef liver	7 ± 1	8 ± 2	0.06 ± 0.001	53	47	15	3.5	0.5
	Pig muscle	7 ± 2	9 ± 0.5	0.07 ± 0.005	84	16	16 ± 2	5.3	0.8
*L. sericata*	Artificial diet	5	5	0.035 ± 0.002	84	16	10	8.4	0.6
	Beef liver	5 ± 0.5	6 ± 0.6	0.035 ± 0.003	84	16	11 ± 1	7.6	0.6
	Pig muscle	5 ± 0.3	5 ± 0.4	0.038 ± 0.003	82	18	10 ± 1	8.2	0.6
*S. argyrostoma*	Artificial diet	5	8	0.09 ± 0.002	78	22	13 ± 1	6.0	1.4
	Beef liver	5	8 ± 1	0.09	78	22	13 ± 1	6.0	1.4
	Pig muscle	5	8	0.09 ± 0.003	81	19	13	6.2	1.4

^1^Growth index = % adult formation/developmental period. ^2^Index of adequacy = (pupal weight/larval period) x % adult formation. SE, standard error.

In *H. illucens* and *C. vicina*, there were differences in the larval and pupal duration between larvae reared on the artificial diet and those reared on beef liver or pig muscle. Conversely, the development of *S. argyrostoma, L. sericata*, and *M. domestica* demonstrated similar performance on natural and artificial diets under laboratory conditions.

For most of the tested species, the growth index revealed that the performance of the artificial diet was better than that of the other natural substrates. Only *M. domestica* showed a high growth index in the pig muscle diet. The adequacy indices for all species were higher than or similar to those for insects reared on other natural substrates. *C. vicina* showed an adequacy index slightly higher than that of the artificial diet (0.8) when raised in pig muscle.

**Figure 1:**
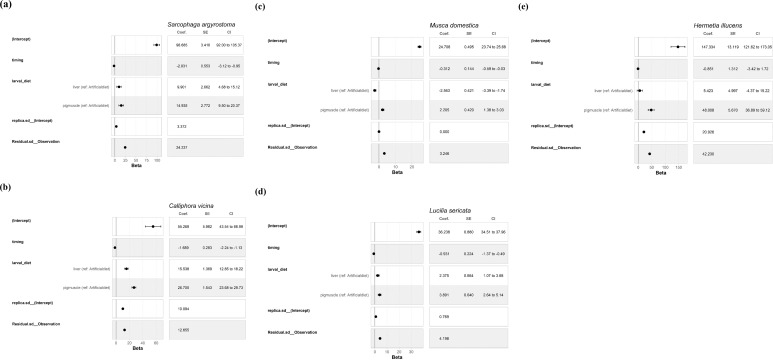
Results of the model for the effects of time and diet on pupal weight. *Sarcophaga argyrostoma* **(a)**, *Calliphora vicina* **(b)**, *Musca domestica* **(c)** *Lucilia sericata* **(d)**, and *Hermetia illucens* **(e)**. Intercept: This represents the estimated mean pupal weight (× 1,000) for the reference group (baseline larval diet, at time = 0).

The results of the models for the effects of time and diet on pupal weight for different species are presented in a coefficient table, accompanied by a forest plot in **Figure 1**, along with a comprehensive description and guide to interpreting the results.

For all bred species, an increase of one unit in time was associated with a decrease in pupal weight. The figures show that larvae reared on the liver and muscle diet produced heavier pupae than those reared on the artificial diet. The only exception was *M. domestica*, for which larvae raised on liver produced lighter pupae than those raised on the artificial diet. However, *H. illucens* larvae raised on the liver diet produced pupae that were 5.423 points heavier than those raised on the baseline diet. However, the plausible range varied from -4.39 to 15.18, indicating an effect that may be negative, close to zero, or positive. This suggested that there was uncertainty regarding whether a liver diet was beneficial. For *S. argyrostoma, M. domestica*, and *L. sericata*, the replicate effect was very small, and the compatibility interval was zero, suggesting that the replicates contributed little or no systematic variability in pupal weight. For the other species, the different experimental replicates showed clear differences in mean pupal weight, indicating that the replicates were a significant source of variability.

## 4 Discussion

In recent years, several publications have focused on food quality as an additional biotic factor for plasticity in larval growth and development. Clark et al. (29) observed that larvae of the blow fly *Lucilia sericata* grew faster when reared on lung and heart in comparison to liver. El-Moaty and Kheirallah (30) presented a similar amount of variation for the same species when fed with brain, lung, liver, kidney, heart, meat, or intestine. Bernhardt et al. (31) investigated the larval growth and development of the blowfly *C. vicina* on human muscle tissue in comparison with common porcine tissues. Their results focused only on larval growth using natural substrates and did not consider the entire development cycle.

Several artificial diet formulations are available for different flies (32–36), especially in the field of forensic science.

The results of this study clearly showed that larval growth and development were supported by the diets tested, indicating that an artificial diet may effectively replace other protein-based substrates, such as liver or muscle, for rearing saprophagous flies.

The adequacy index of the artificial diet was similar to that of the other diets tested, indicating an optimum energetic and nutritional contribution. The percentage of adults fed the three diets that emerged was greater than 75%, demonstrating adaptation to the set of nutritional and physical–environmental conditions prevalent in the laboratory. However, it is worth emphasizing that greater emergence success was observed on artificial diets and in pig muscle than in beef liver. Indeed, for larvae reared on artificial diet, the adult emergence rate was between 78-98%, for larvae reared on pork muscle between 68-98% and for liver the range was between 32-92%. Some nutritional components of the artificial diet (proteins and fats provided by milk powder, casein, and yeast) may be more appropriate for nurturing and developing competent larvae and facilitating their survival. This pattern was also observed in another study (37). Pupal weight is a critical factor determining insect survivorship (38). Previous studies of *L. sericata* and *Cochliomyia hominivorax* (Diptera: Calliphoridae) have demonstrated the importance of pupal weight as a measure of colony fitness. For example, the primary screwworm pupates successfully only 3.9% of the time when the larval weight ranges between 21 and 25 mg but shows 100% successful pupation when the larvae weigh 56–60 mg (39). The pupal weight of *L. sericata* was helpful in differentiating the effect of diet on development, with the fresh beef liver diet producing the weighty pupae, the blood–yeast agar diet producing the smallest pupae, and the decomposed beef liver diet and powdered beef liver diet producing intermediate-sized pupae (40).

In the last days of larval development, there is an accumulation of nutritional reserves in the larval body, which can be used during the subsequent development phases (41). These reserves accumulate in the adipose tissues. Therefore, the different levels of fat content in the diet can influence the size of both the larval and pupal body. In addition, effects on development times can also occur, leading the larvae to migrate and pupate early, leading to a misleading estimate of the PMI (42).

We found that the pupal weight was different between the three diets tested, except for *S. argyrostoma*, but this had no effect on the death rate. When reared on beef liver, the death rates were higher for *H. illucens*, *M. domestica*, and *C. vicina*. This may be explained by differences in the protein composition of the diets and the fact that *H. illucens* requires saccharides (43).

An evaluation of developmental time showed that the artificial diet did not cause differences in the egg-to-adult period, except for *M. domestica* and *H. illucens*. This confirmed the results of other studies in which the mass rearing of houseflies was possible on plant-based substrates consisting of 50% wheat bran, 30% alfalfa meal, and 20% corn meal. These substrates produced the higher survival-to-pupation ratios and greater pupal weights compared to those produced by animal waste (e.g. dairy, swine, or poultry manure) (44). The black soldier fly, *H. illucens*, although capable, like blow flies, of colonizing carrion, is considered a detritivore because its larvae feed on a variety of resources, including plant waste and restaurant food waste. When the larvae of this species are not reared on plant-based substances, they can extend their developmental period because of a lack of sufficient nutrients to survive through pupation and adult emergence.

Cook et al. (45) demonstrated that plant-based substrates are unsuitable for the mass rearing of *C. vicina*. *C. vicina* develops very slowly, even when soy flour is supplemented with other meat-based ingredients. Our study partially contradicts this result, showing that a diet supplemented with soy flour and the correct percentage of animal proteins represents a good alternative for the mass rearing of necrophagous insects.

Our data confirmed that all ingredients and their concentrations were likely indispensable for the growth of the five Dipteran species tested under the experimental conditions applied.

Fly rearing contributes to many aspects of our daily lives; for example, the production of human food and feed products, medicinal treatments for difficult-to-heal wounds, the transformation of large quantities of organic wastes that we generate daily into valuable products for the cosmetic or animal feed industries, the pollination of crops and their protection from pests (thereby avoiding the use of pesticides), and as evidence in criminal cases in the case of insects of forensic importance. Different diets affect the developmental time, size, and adult emergence of species of forensic importance (7, 8). This prompted us to prepare a standard diet on which they could be easily reared for research and other studies related to forensic entomology.

The artificial diet we developed was found to be most suitable for use in the laboratory. Its most relevant advantages are: 1) it has much less odor than liver or pig muscle diets. In fact, the gelling agent may have absorbed much of the metabolic waste, thereby reducing the amount of odor emitted during larval rearing; 2) it is simple to prepare and handle. All dry ingredients used to prepare the diet are commercially available from the indicated vendors; 3) it is less expensive to purchase in bulk and can be stored in a cool and dry place for an extended period, with no apparent loss of nutritional quality. However, our artificial diet has some disadvantages that need to be addressed. Although the artificial diet guarantees growth rates only like those of natural diets, thus offering advantages for forensic use, this also becomes a disadvantage for mass insect production. Our artificial diet likely does not guarantee the creation of populations dissimilar from natural ones, as it eliminates the differences that may exist in a natural population and influence development and evolution. Indeed, a short development period, high fertility, the laying of larger eggs highly enriched with nutrients, and the hatching rate are the proper parameters for determining a mass-rearing system, as well as key characteristics for evaluating artificial diets (46).

It has long been recognized that diets varying in nutritional composition influence insect development, reproduction, and longevity (47). Beyond providing sustenance and essential nutrients, the food ingested by insects supports the growth of numerous gut bacteria, resulting in a diverse and relatively stable microbial community (48). In this study, non-sterile diets were used to avoid alterations in the nutritional quality and physical properties of the diet components that can result from sterilization. This approach is aimed at preserving the microbial integrity of the artificial diet. Furthermore, as highlighted by Ponton et al. (49), diet quality and access to specific nutrients can affect both insect development and immune system performance. Inadequate nutrition during the larval stage may lead to a significantly weakened immune response. Future research will explore the potential effects of this artificial diet on the reproductive capacity of emerged adults. This strategy will contribute to the development of an artificial diet suitable not only for forensic entomology but also for broader applications, including the mass rearing of necrophagous and scavenging flies.

## Data Availability

The raw data supporting the conclusions of this article will be made available by the authors, without undue reservation.
